# Outcomes in older kidney recipients from older donors: A propensity score analysis

**DOI:** 10.3389/fneph.2022.1034182

**Published:** 2022-10-20

**Authors:** Elena Cuadrado-Payán, Enrique Montagud-Marrahi, Joaquim Casals-Urquiza, Jimena del Risco-Zevallos, Diana Rodríguez-Espinosa, Judit Cacho, Carolt Arana, David Cucchiari, Pedro Ventura-Aguiar, Ignacio Revuelta, Gaston J. Piñeiro, Nuria Esforzado, Frederic Cofan, Elisenda Bañon-Maneus, Josep M. Campistol, Federico Oppenheimer, Josep-Vicens Torregrosa, Fritz Diekmann

**Affiliations:** ^1^ Department of Nephrology and Kidney Transplantation, Hospital Clinic Barcelona, Barcelona, Spain; ^2^ Laboratori Experimental de Nefrologia i Trasplantament (LENIT), Institut d’Investigacions Biomèdiques August Pi iSunyer (IDIBAPS), Barcelona, Spain; ^3^ Red de Investigación Renal (REDINREN), Madrid, Spain

**Keywords:** older donors, older recipients, graft survival, recipient survival, propensity score, kidney transplantation

## Abstract

**Background:**

The age of patients referred for kidney transplantation has increased progressively. However, the precise influence of age on transplant outcomes is controversial.

**Methods:**

Etrospective study in which graft and recipient survival were assessed in a cohort of ≥75 years old kidney recipients and compared with a contemporary younger one aged 60-65 years through a propensity score analysis.

**Results:**

We included 106 recipients between 60-65 and 57 patients of ≥75 years old with a median follow-up of 31 [13-54] months. Unadjusted one- and five-year recipient survival did not significantly differ between the older (91% and 74%) and the younger group (95% and 82%, P=0.06). In the IPTW weighted Cox regression analysis, recipient age was not associated with an increased risk of death (HR 1.88 95%CI [0.81-4.37], P=0.14). Unadjusted one- and five-year death-censored graft survival did not significantly differ between both groups (96% and 83% for the older and 99% and 89% for the younger group, respectively, P=0.08). After IPTW weighted Cox Regression analysis, recipient age ≥75 years was no associated with an increased risk of graft loss (HR 1.95, 95%CI [0.65-5.82], P=0.23).

**Conclusions:**

These results suggest that recipient age should not be considered itself as an absolute contraindication for kidney transplant

## Introduction

Kidney transplantation has demonstrated to improve the survival of patients with end-stage kidney disease (ESKD) compared to those who remain on dialysis (1, 2). During the last decade life expectancy has increased in patients with ESKD on dialysis and, therefore, in those who are referred for kidney transplant and included on the waiting list, as well as kidney donors (3–6). In Spain, approximately 15% of transplant recipients and 20% of kidney donors are> 70 years old, a percentage that has increased in recent years (3, 4). The influence of the recipient age on patient and graft outcomes after kidney transplant is an increasingly important issue given the aging of patients who are candidates for a kidney transplant (3, 6–9).

The benefit of kidney transplantation in elderly patients is controversial, although there is increasing evidence that age should not be considered as an absolute contraindication for kidney transplant (8–11). In fact, some studies have shown that kidney transplantation, even in population of>80 years old, represents a benefit in terms of survival compared to remaining on dialysis (8, 10). Similarly, recent studies have demonstrated the viability and safety of kidneys from octogenarian donors (12). Nevertheless, an important aspect that difficult to draw solid conclusions from studies that assess the impact of the recipient age on recipient and graft outcomes is the frequent overlap between the age ranges of the compared groups, which prevents a clear separation between the outcomes of each one of them (8, 11).

The aim of the present study was to evaluate the impact of the recipient age on patient and kidney graft outcomes in a cohort of ≥ 75 years-old patients, but using as a reference a cohort of recipients with an age difference of at least 10 years (60-65 years). Furthermore, since the majority of older recipients received a kidney from an elderly donor, the influence of the donor’s age was also evaluated.

## Materials and methods

### Study design

Single-center, longitudinal and retrospective study in which kidney transplant recipients between 60 and 65 and ≥ 75 years old performed in our center from January 1st, 2010 to June 30th 2019 were included. Follow-up and data collection were performed until June 30th, 2020. The study protocol was conducted in accordance with the principles of the Declaration of Helsinki and approved by the Research Ethics Committee of the Hospital Clinic.

### Patient population

Demographic, clinical, analytical and immunological data were collected from both donors and recipients and outcomes from the older group (≥ 75 years) with respect to the younger one (60-65 years) were compared. Pre-transplant patient assessment included cardiologic evaluation (electrocardiogram, echocardiography and a stress test for ischemia detection), and CT-scan of splanchnic and iliac vessels. Immunological workup included complement dependent cytotoxicity (CDC), panel reactive antibodies (PRA) and solid phase Luminex^®^ screening. Solid phase single bead antigen was performed in the presence of a positive class I and/or II Luminex^®^ screening. Expanded Criteria Donor (ECD) was defined as donor age ≥ 60 years or 50 to 59 years and two of the following: [1] Cause of death is cerebrovascular accident; [2] pre-existing history of systemic hypertension; and [3] terminal serum creatinine >1.5 mg/dl (13).

### Outcomes definition

Primary outcomes included recipient and kidney graft survival at one and five years after transplantation. As secondary outcomes, we evaluated one-year biopsy-proven acute rejection (BPAR), new onset neoplasms, the rate of post-transplant infection during follow-up, kidney delayed graft function (DGF), which was defined as the need for at least one session of hemodialysis during the first week following kidney transplantation, as well as death-censored kidney graft failure, which was defined as return to dialysis or re-transplantation.

### Immunosuppression

Induction immunosuppression therapy was used in all patients. Induction therapy consisted in two doses of anti-IL2 monoclonal antibody (basiliximab) of 20 mg at day 0 and at day +4 after surgery or rabbit anti-human lymphocytes polyclonal antibodies (either Thymoglobulin^®^ 1,25mg/Kg/day or ATG^®^ 2,5mg/Kg/day) for 5 consecutive days in immunological risk recipients (defined as a cPRA> 25% or/and ≥ 1 previous kidney transplant lost because an immunological etiology) and in kidney transplants from a Donor After Circulatory Death (DCD). Maintenance immunosuppression protocol was based on triple therapy with tacrolimus, mycophenolate or mTOR inhibitors, and steroids (methylprednisolone in the immediate post-transplant period, followed by oral prednisone).

### Statistical analysis

The normal distribution of continuous variables was evaluated with the Kolmogorov-Smirnov test. Data are presented as mean (standard deviation, SD) for parametric variables and median (interquartile range [IQR]) for the non-parametric ones. The corresponding tests used were T-test or Mann-Whitney U test as appropriated. Categorical data were compared using the Chi-Square test.

Inverse probability of treatment weighting (IPTW) was used to account for covariate imbalance between older and younger recipients. IPTW was estimated from a propensity score from a logistic regression model to be assigned to the older and younger group. The model included: donor age, transplantation from DCD donors, cold ischemia time, dialysis before kidney transplant, diabetes mellitus (DM) before kidney transplant, ECD, Major Adverse Cardiovascular Event (MACE) before transplant, induction and maintenance immunosuppression.

A stabilized weighting method was performed by multiplying the IPTW by the proportion of recipients with ≥ 75 and 60-65 years old. Check for adequate balance of covariates after IPTW analyses was performed by calculation of standardized differences and an absolute difference greater than 0.1 represented a meaningful imbalance (14, 15). All subsequent analyses were performed on the weighted, covariate-balanced population. Kaplan-Meier was used to estimate patient and graft survival and compared using log-rank test. Binominal logistic regression was used to calculate odds ratio, and Cox proportional regression was performed to estimate patient and graft hazards.

Statistical analysis was conducted using IBM SPSS Statistics 22.0 (SPSS, Inc; Chicago, Illinois) software for Windows. Graphical representation of Kaplan-Meier survival curveswas designed with GraphPad v.5 (GraphPad Software, La Jolla, CA, US). All tests were two-tailed and the significance level was defined as a P value <0.05.

## Results

### Baseline characteristics

A total of 163 kidney recipients were included. Of these, 106 were between 60 to 65 years old (mean age 63 ± 1.7 years) and 57 were ≥75 years (78 ± 2.1 years). Median follow-up was 31 [13-54] months.

Prevalence of pre-transplant recipient comorbidities as dyslipidemia, smoking, hypertension and diabetes were not significantly different between both groups, as well as prevalence of Ischemic Heart Disease (IHD), Cerebrovascular Accident (CVA), dialysis modality and dialysis vintage (**Table 1**).

**Table 1:** Baseline characteristics of the included patients.

	60–65 years (n=106)	≥ 75 years (n = 57)	*P*
Age at KT (years)	63.12 ± 1.68	77.72 ± 2.10	< 0.0001
Gender (Male)	59 (56)	39 (68)	0.11
BMI (Kg/m^2^)	26.24 ± 4.24	25.70 ± 3.81	0.48
DM	49 (46)	20 (35)	0.19
Dyslipidemia	57 (54)	24 (42)	0.19
MACE before KT (any)	21 (20)	16 (28)	0.23
IHD	19 (18)	13 (23)	0.54
CVA	7 (6)	4 (7)	0.92
Smoking	14 (13)	4 (7)	0.23
Hypertension	95 (90)	49 (86)	0.49
CKD etiology			0.001
Nephroangioesclerosis	11 (10)	7 (12)	
Diabetic Nephropathy	30 (28)	2 (4)	
Glomerular disease	14 (13)	7 (12)	
ADPKD	14 (13)	7 (12)	
Urological	8 (8)	2 (4)	
Unknown	23 (22)	30 (52)	
Other	6 (6)	2 (4)	
Dialysis vintage (months)	31 [21-59]	26 [16-49]	0.24
Dialysis type			0.15
Pre-dialysis	4 (4)	3 (5)	
Hemodialysis	87 (82)	46 (81)	
Peritoneal dialysis	15 (14)	8 (14)	
Previous ≥1 KT	25 (24)	8 (14)	0.15
cPRA> 50%	32 (31)	9 (16)	0.03
Total HLA mismatches	4 ± 1.05	4 ± 1.16	0.56
Donor sex (male)	41 (39)	29 (51)	0.13
Donor age (years)	64.92 ± 10.23	76.11 ± 8.12	< 0.0001
DDKT type			0.10
DBD	58 (55)	39 (68)	
DCD	47 (45)	18 (32)	
ECD	88 (83)	55 (97)	0.01
CIT (h)	15.04 ± 6.08	15.50 ± 5.17	0.66
Induction IS			0.01
Thymoglobulin	76 (72)	29 (51)	
Basiliximab	30 (28)	28 (49)	
Maintenance IS			0.48
PDN + TAC + MMF	47 (44)	22 (38)	
PDN + TAC + mTORi	59 (56)	35 (61)	

Data are expressed as mean ± SD, median [IQR] or n (%) unless otherwise indicated. KT, kidney transplantation; BMI, Body Mass Index; DM, Diabetes Mellitus; IHD, Ischemic Heart Disease; CVA, Cerebrovascular Accident; CKD, Chronic Kidney Disease; ADPKD, Autosomal Dominant Polycystic Kidney Disease; HLA, Human Leukocyte Antigen; DDKT, Deceased Donor Kidney Transplantation; DBD, Donor after Brain Death; DCD, Donor after Circulatory Death; ECD, Expanded Criteria Donor; CIT, Cold Ischemia Time; IS, immunosuppression; PDN, Prednisone; TAC, Tacrolimus; MMF, mycophenolate; mTORi, mTOR inhibitor.

In the ≥ 75 years old group, donors were older (76.11 ± 8.12 vs. 64.92 ± 10.23 years for the older and younger group, respectively, P<0.0001) andwere more frequently ECD (97% vs. 83% in the older and the younger group, respectively, P=0.01) (**Table 1**).

**Table 1** summarizes the main baseline characteristics of the included patients. Any of the recipients included in the study had pre-formed DSAs.

After IPTW adjustment, no significant differences in donor and recipient characteristics were observed (except for recipient age). **Supplementary Tables S1** and **S2** summarize main baseline characteristics of the weighted, balanced population.

### Recipient outcomes

**Figure 1:**
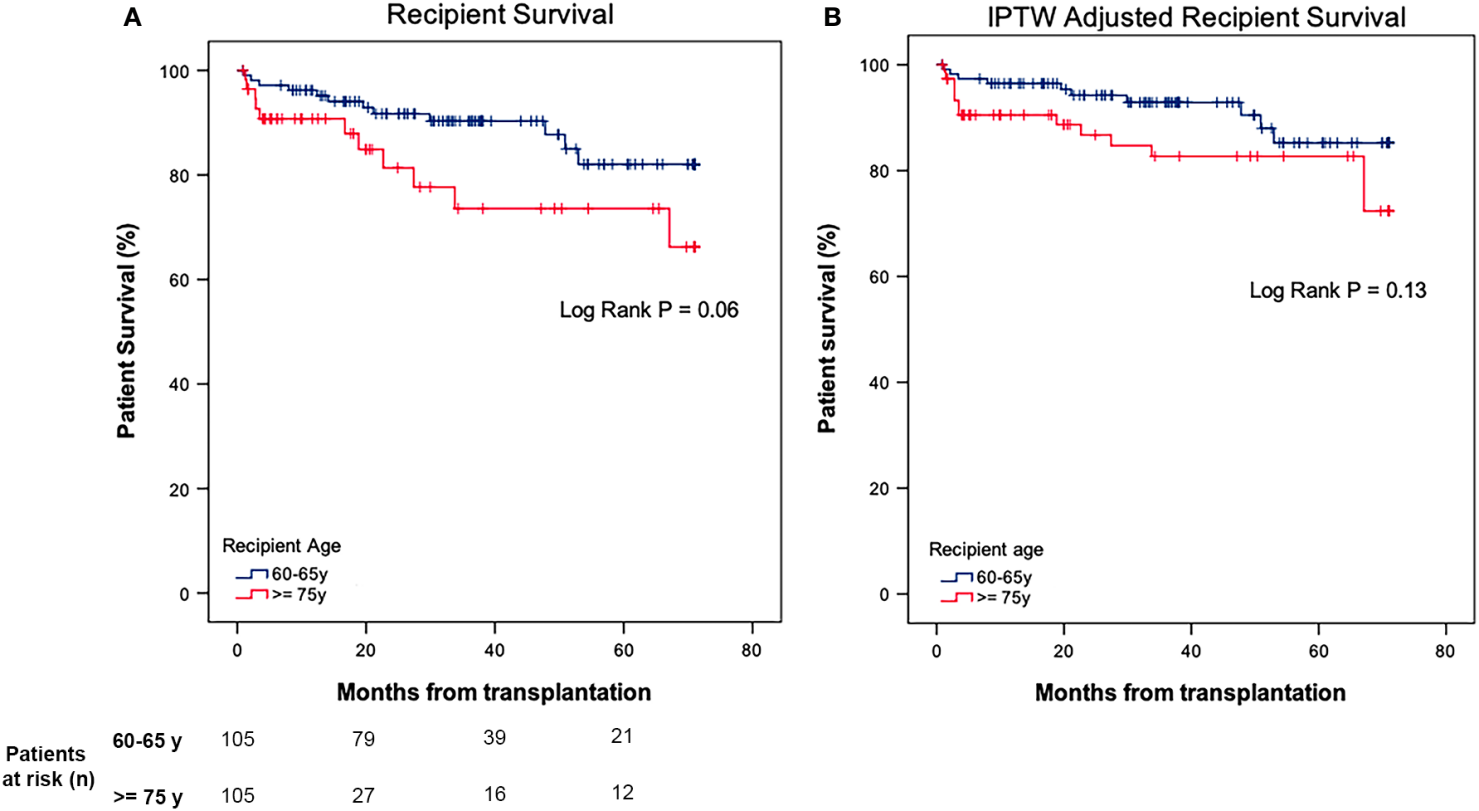
**(A)** Recipient survival after kidney transplantation in 60-65 vs ≥ 75 years old recipients. **(B)** Recipient survival after kidney transplantation in 60-65 vs ≥ 75 years old recipients after IPTW weighting.

Recipient survival was not significantly different for recipients of ≥75 years when compared to those between 60-65 years, with 1- and 5-year recipient survival of 95% and 82% for the group 60-65 years (mean survival time of 63.75 ± 1.95, 95% CI [59.93 - 67.58] years), and 91% and 74% for the group of ≥75 years (mean survival time of 56.41 ± 3.98, 95% CI [48.59 - 64.21] years), respectively (P = 0.06) (**Figure 1A**). These results were also observed after IPTW weighting with 1- and 5-year recipient survival of 97% and 85% for the group 60-65 years (IPTW adjusted mean survival time of 65.37 ± 1.68, 95% CI [62.07-68.67] years), and 91% and 83% for the group of ≥75 years (IPTW adjusted mean survival time of 60.57 ± 2.78, 95% CI [55.13 - 66.03] years), respectively (P = 0.13) (**Figure 1B**). There were no significant differences between both age groups for cause of death (P = 0.22): infection was the main cause of death (especially in the older group), with 4 (33%) and 8 cases (73%) in the 60-65 and ≥ 75 years group, respectively (**Supplementary Table S3**).

During follow-up, 7 (7%) and 2 patients (4%) in the younger and older group developed *de novo* malignancies, respectively. The most frequent malignancies were solid organ malignancies (5%, 5 patients) in the group of 60-65 years, and solid organ and hematological malignancies ≥ 75 years group (1% for each) (**Table 2**).

**Table 2:** Recipient and Graft Outcomes at follow up.

	60–65 years (n=106)	≥ 75 years (n = 57)	*P*
1-year SCr (mg/dL)	1.78 ± 0.88	1.60 ± 0.58	0.23
1-year eGFR (mL/min/1.73m^2^)	42.80 ± 16.31	42.41 ± 15.87	0.76
1-year proteinuria (mg/g)	208 [98 - 522]	346 [171 - 627]	0.06
SCr at last FU (mg/dL)	1.70 ± 0.63	1.84 ± 0.69	0.51
eGFR at last FU (mL/min/1.73m^2^)	44.14 ± 15.34	37.13 ± 17.75	0.54
1-year post-KT infection (any)	35 (33)	19 (35)	0.78
CMV replication	45 (43)	28 (49)	0.26
1-year biopsy chronicity scores (median [range)]			
ci	1 ± 0.73	1 ± 0.69	0.46
ct	1 ± 0.56	1± 0.66	0.22
cv	1 ± 0.79	1 ± 0.86	0.53
cg	0 ± 0.27	0 ± 0.22	0.66
1-year BPAR	17 (16)	9 (16)	0.97
TCMR	11 (10)	5 (9)	0.76
ABMR	7 (7)	6 (11)	0.37
Delayed Graft Function	34 (32)	14 (25)	0.32
BK nephropathy	3 (3)	2 (4)	0.81
Post-KT Neoplasm (any)	7 (7)	2 (4)	0.41
Solid organ neoplasm	5 (5)	1 (2)	
Haematological	2 (2)	1 (2)	

Data are expressed as mean ± SD, median [IQR] or n (%) unless otherwise indicated. SCr, Serum Creatinine; FU, Follow-up; eGFR, estimated Glomerular Filtration Rate; KT, Kidney Transplantation; CMV, cytomegalovirus; cg, transplant glomerulopathy; ci, interstitial fibrosis; ct, tubular atrophy; cv, transplant arteriopathy. BPAR, Biopsy Proven Acute Rejection; TCMR, T-cell Mediated Rejection; ABMR, Antibody Mediated Rejection.

In the Cox regression analysis after IPTW weighting, recipient age ≥75 years was not independently associated with lower recipient survival (HR 1.88 95% CI [0.81-4.37], P = 0.14).

### Graft outcomes

Kidney graft function at one year did not differ significantly between both age groups, with 1-year serum creatinine (SCr) of 1.78 ± 0.88 and 1.60 ± 0.58 mg/dL (P = 0.23), and estimated glomerular filtration rate (eGFR) of 42.80 ± 16.31 and 42.41 ± 15.87 mL/min/1.73m2 for the 60-65 and ≥ 75 years old group, respectively (P = 0.76). At last follow-up, there also were not significant differences in the SCr (1.70 ± 0.63 and 1.84 ± 0.69 mg/dL for the younger and older group, respectively, P = 0.51) or in the eGFR between both age groups (44.14 ± 15.34 vs 37.13 ± 17.75mL/min/1.73m2 for the younger and older group, respectively, P = 0.54) (**Table 2**).

One-year Biopsy-Proven Acute Rejection (BPAR) rate was of 16% in both age groups (17 and 9 cases in the group of 60-65 and ≥ 75 years, respectively), although in the younger one the most frequent was T-Cell Mediated Rejection (TCMR)(10%, 11 cases) and in the older group was Antibody-Mediated Rejection (ABMR) (6%, 11 cases). There were no statistically significant differences in the overall rate or in the rejection type between both groups (P = 0.97) (**Table 2**). No differences were observed in the histological chronicity scores at one year, the rate of BK nephropathy, or the rate of delayed graft function (DGF) after transplantation (**Table 2**).

**Figure 2:**
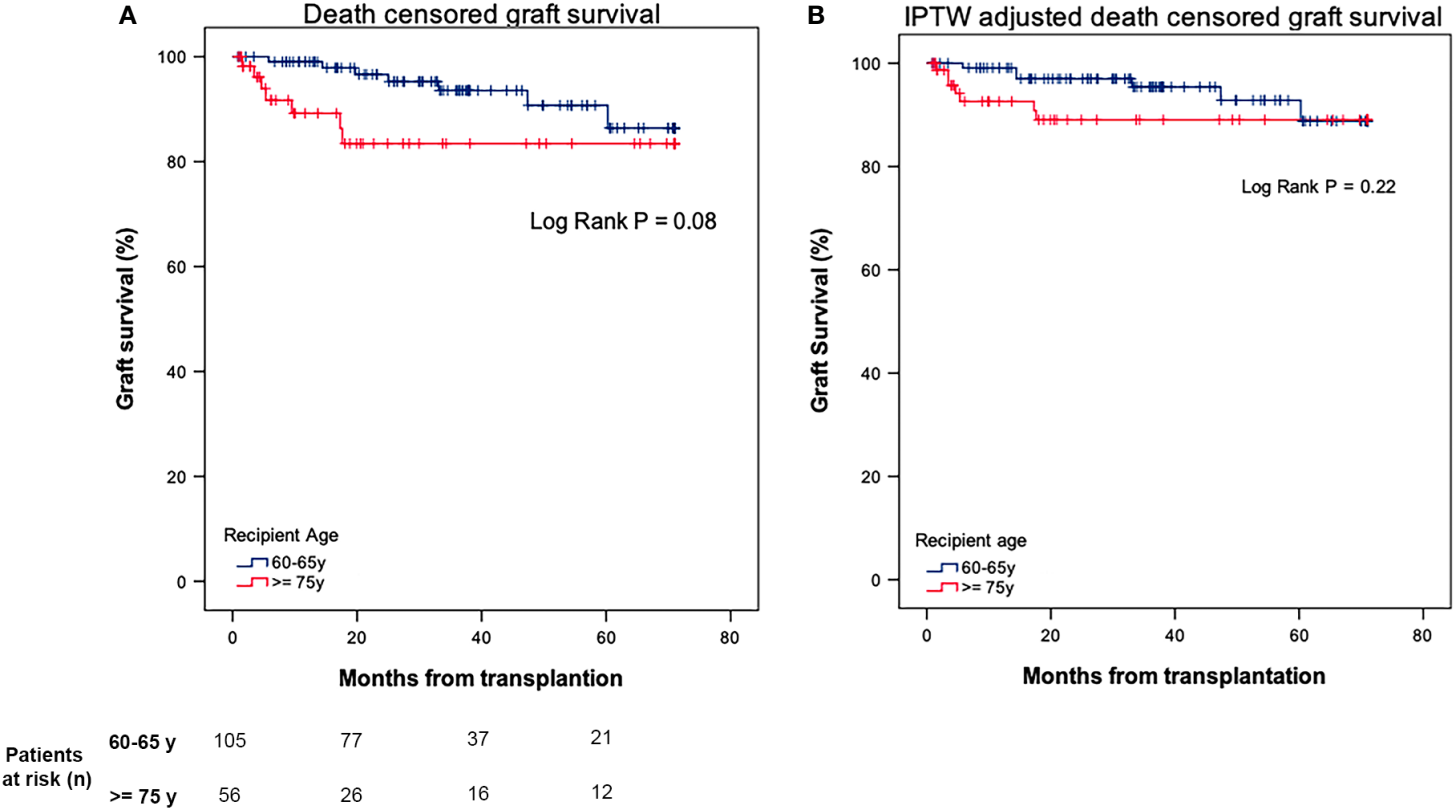
**(A)**, Death-censored graft survival after kidney transplantation in 60-65 vs ≥ 75 years old recipients. **(B)** Death-censored graft survival after kidney transplantation in 60-65 vs ≥ 75 years old recipients after IPTW weighting.

Moving to death-censored graft survival, 1- and 5-year survival rate was of 99% and 89% for the younger group, and 96% and 83% for the older group, respectively, without significant differences between them (P = 0.08) (**Figure 2A**). These results were also observed after IPTW weighting, with a 1- and 5-year survival rate was of 99% and 93% for the younger group, and 93% and 89% for the older group(P = 0.22) (**Figure 2B**).

In the IPTW-adjusted Cox regression analysis, recipient age ≥ 75 years was not associated with an increased risk of graft loss (HR 1.95, 95% CI [0.65 - 5.82], P = 0.23) (**Table 3**).

**Table 3:** Cox regression analysis for patient and death censored graft survival after IPTW weighting.

Variable	HR	95% CI		*P*
		LL	UL	
Patient survival				
Age at KT 60 – 65 years	1.000			
Age at KT ≥ 75 years	1.884	0.81	4.37	0.14
				
Death censored graft survival				
Age at KT 60 – 65 years	1.000			
Age at KT ≥ 75 years	1.947	0.65	5.82	0.23

KT, Kidney Transplantation; HR, Hazard Ratio; LL, Lower Limit; UL, Upper Limit; CI, Confidence Interval.

## Discussion

In the present study we analyzed recipient and kidney graft survival in a cohort of older recipients with ≥75 years using a stabilized IPTW weighting method for donor and recipient covariates, comparing their outcomes with those of a cohort of recipients with 60-65 years and, thus, with at least a 10-year difference in recipient age between both groups. In summary, we observed that the recipient and graft survival up to 5 years after transplantation was not significantly different between both age groups.

Kidney transplantation has demonstrated to be the treatment of choice for end-stage kidney disease (ESKD) (1, 6, 16). However, the aging of the general population has led to a notable increase in the mean age of patients with ESKD, which, secondarily, has significantly risen the mean age of patients who are referred to evaluation for kidney transplantation (3, 5, 6). The potential negative influence of the recipient age on patient and graft survival has led to consider the age as an isolated criterion to accept or discard a candidate for a kidney transplant, thus depriving the patient of it smedium- and long-term benefits (2, 7, 8, 10–12, 17–19). During the last years, different studies have been published evaluating the influence of recipient age on kidney transplant outcomes with controversial results, although available information which suggests that age is not independently associated with worse graft and recipient survival is increasing (7, 8, 10, 11, 20).

In 2016, Lønning et al. (10) compared a cohort of 35 of recipients > 79 years old with a younger one of 364 patients aged 70-79 years concluding that age was not independently associated with higher recipient mortality or higher rate of kidney graft failure. Similar results were recently reported by Cabrera et al. in a cohort of 138 recipients of ≥75 years (8). Nevertheless, one common aspect to highlight in most studies is the frequent and potential overlap between the age ranges of the two compared groups, which does not allow to completely separate both cohorts and, as a consequence, would make it difficult to draw solid conclusions about the influence of age on recipient and graft outcomes in older patients compared to truly younger recipients (7, 10, 21). In fact, it should be noted that the differences in outcomes reported by Lønning et al. would be tempered by the relatively similar ages of the included patients, which are significantly different to the patients in our study (10). Thus, we decided to analyze recipient and graft outcomes in a cohort of patients ≥75 years but taking as a reference a cohort of recipients between 60-65 years in order to establish a clear age separation with at least 10 years between both groups. Recipient survival in the older group was 91% and 74% at 1 and 5 years after transplantation, which is similar to that already published in previous series (7–10, 21). However, the 5 years patient survival was higher than that reported by Lønning et al. (10), although in that study the 68% of the octogenarian group were transplanted before 2010, which contrasts with our cohort. As in previous studies, the main cause of death in both groups were infections, especially in older recipients (22, 23), and we did not identify differences in terms of transplant-associated complications.

After IPTW weighing for donor and recipient covariates, age was not independently associated with an increased risk of recipient death, a result which is in line with some already published, although using as reference groups patients with a closer age range to the evaluated one (10). Our results contrast with those published by Huang et al. in 2010 (11). They analyzed the outcomes after kidney transplantation in a large cohort of recipients of 70-79 and ≥80 years old using as reference a younger one of 60-69 years. They conclude that recipient age was associated with a decreased recipient survival, even when adjusting for confounding factors. Nevertheless, in this study kidney transplants were performed between 2000 and 2008, while in our case all patients were transplanted after 2010. Since the influence of transplant era in recipient and graft survival is well established, we speculate that this temporary gap could, at least in part, justify these differences (10, 24).

Death-censored graft survival at 1 and 5 years was not significantly different when compared to the reference younger cohort, and was also similar to that previously reported (8, 10, 11). Graft function at 1 year and at the end of the follow up was also similar between both groups. After IPTW adjustment, the age ≥ 75 years was not independently associated with an increased risk of graft loss. Importantly, mean donor age was as higher as 76 years and, as expected, there was an important percentage of ECD in the older group when compared to the younger cohort. Although in the present study the independent association between donor age and ECD with recipient and graft outcomes could have not been assessed (since IPTW analysis has taken into account both variables for weighting), the higher frequency of older donors and ECD in the older group suggests that recipient age itself but donor characteristics would be important factors which should guide kidney transplant in older recipients to avoid an excess mortality after transplantation, an issue that has been already suggested elsewhere (8, 9). Previous systematic reviews suggested ECD kidneys may be better prioritized for older recipients by ignoring immunology-based allocation. Using this strategy, the Eurotransplant Senior programme have shown favorable 5-year outcomes using ECD kidneys in older recipients (25). Also, in our country, we carry out a similar strategy in addition to ensuring short ischemia times, perform a preimplantation biopsy in such donors and place the kidneys on a hypothermic machine perfusion.

The authors acknowledge that the study has some limitations that should be taken into account when extrapolating the results. This is a retrospective and single-centre study in which a cohort of older recipients that has been considered suitable for kidney transplantation has been compared with a younger group. This could have induced a selection bias since the included recipients in the waiting list have probably been patients with only few comorbidities and a good condition to be transplanted, as well as the donor has been selected according to the recipient risk to minimize the consequences of a non-functioning kidney graft. Nevertheless, the IPTW method partially reduces the effect of this potential selection bias by approaching baseline characteristics between both groups. Another limitation to take into account is the relatively small sample size of older recipients with the short number of events that make necessary to be cautious with the conclusions, especially when extrapolating to other populations to avoid bias.

Despite these limitations, we believe that our study provides more evidence about the impact of the recipient age in kidney transplantation by comparing older recipients with a younger cohort whose age range is clearly separated from its older counterparts and showing the lower mortality we have nowadays in this kidney recipients.

In conclusion, in the present study we suggest that recipient age by itself should not be a criterion to contraindicate kidney transplantation. A complete evaluation of both donor and recipient (specially focusing on recipient comorbidities) should guide kidney transplant indication in older recipients. Thus, with a judicious selection of both the recipient and the donor, kidney transplantation can be safely performed in elderly recipients.

## Data availability statement

The raw data supporting the conclusions of this article will be made available by the authors, without undue reservation.

## Ethics statement

Ethical review and approval was not required for the study of human participants in accordance with the local legislation and institutional requirements. Written informed consent from the patients/participants or patients/participants legal guardian/next of kin was not required to participate in this study in accordance with the national legislation and the institutional requirements.

## Author contributions

EC-P analyzed, interpreted the data and prepared the manuscript. EM-M collected, analyzed, and interpreted the data and prepared the manuscript. JC-U collected, analyzed and interpreted the data. JR-Z collected, analyzed and interpreted the data. DR-E collected, analyzed and interpreted the data, JC collected, analyzed and interpreted the data, CA collected, analyzed and interpreted the data, DC collected the data and critically revised the manuscript. PV-A interpreted the data and critically revised the manuscript. IR interpreted the data and critically revised the manuscript. GP interpreted the data and critically revised the manuscript. NE interpreted the data and critically revised the manuscript. FC interpreted the data and critically revised the manuscript. JU critically revised the manuscript. JMC. interpreted the data and critically revised the manuscript. FO interpreted the data and critically revised the manuscript. J-VT analyzed and interpreted the data and prepared the manuscript. FD interpreted the data and critically revised the manuscript. All authors contributed to the article and approved the submitted version.

## Conflict of interest

The authors declare that the research was conducted in the absence of any commercial or financial relationships that could be construed as a potential conflict of interest.

## Publisher’s note

All claims expressed in this article are solely those of the authors and do not necessarily represent those of their affiliated organizations, or those of the publisher, the editors and the reviewers. Any product that may be evaluated in this article, or claim that may be made by its manufacturer, is not guaranteed or endorsed by the publisher.
