# Supplementary material for: Outcomes in older kidney recipients from older donors: A propensity score analysis
Source: Front Nephrol. 2022 Oct 20;2:1034182. doi: 10.3389/fneph.2022.1034182 (PMC10479569; doi:10.3389/fneph.2022.1034182)
Supplement: Supplementary file 1 [file DataSheet_1.docx]

Supplementary Material

# Supplementary Figures and Tables

Table S1 – Standardized differences before and after IPTW adjustment

|  | Before IPTW adjustment | After IPTW adjustment |
| --- | --- | --- |
| DM | - 0.060 | - 0.090 |
| MACE before KT (any) | 0.113 | - 0.035 |
| Dialysis before KT (yes) | - 0.095 | 0.087 |
| Donor age | 1.143 | 0.095 |
| ECD | 0.328 | 0.085 |
| CIT | 0.082 | - 0.091 |
| Induction IS |  |  |
| Thymoglobulin | - 0.356 | 0.092 |
| Basiliximab | 0.356 | - 0.092 |
| Maintenance IS |  |  |
| PDN + TAC + MMF | - 0.215 | 0.076 |
| PDN + TAC + mTORi | 0.215 | - 0.066 |

IPTW, Inverse probability of treatment weighting; DM, Diabetes Mellitus; MACE, Major Adverse Cardiovascular Event; KT, Kidney Transplantation; ECD, Expanded Criteria Donor; CIT, Cold Ischemia Time; IS, Immunosuppression; PDN, Prednisone; TAC, Tacrolimus; mTORi, mTOR inhibitors. MMF, Mycophenolate.

Table S2 - Baseline characteristics in the weighted, balanced population.

|  | 60–65 years  (n = 94) | ≥ 75 years  (n = 64) | *P* |
| --- | --- | --- | --- |
| Age at KT (years) | 63.14 ± 1.63 | 77.59 ± 1.76 | < 0.0001 |
| Gender (Male) | 57 (61) | 33 (52) | 0.26 |
| BMI (Kg/m^2^) | 25.84 ± 4.04 | 24.68 ± 3.95 | 0.10 |
| DM | 41 (44) | 26 (41) | 0.71 |
| Dyslipidemia | 48 (51) | 32 (50) | 0.90 |
| MACE before KT (any) | 21 (22) | 12 (19) | 0.55 |
| Hypertension | 84 (90) | 59 (91) | 0.93 |
| Dialysis vintage (months) | 42 [25 - 69] | 48 [16 - 170] | 0.41 |
| Dialysis type |  |  | 0.39 |
| Pre-dialysis | 3 (3) | 1 (2) |  |
| Hemodialysis | 78 (83) | 58 (91) |  |
| Peritoneal dialysis | 13 (14) | 5 (7) |  |
| Previous ≥1 KT | 23 (25) | 22 (34) | 0.17 |
| cPRA > 50 % | 26 (29) | 28 (43) | 0.07 |
| Total HLA mismatches | 4.25 ± 1.04 | 4.04 ± 1.12 | 0.25 |
| Donor sex (male) | 37 (39) | 16 (25) | 0.06 |
| Donor age (years) | 67.32 ± 10.37 | 66.87 ± 8.78 | 0.78 |
| DDKT type |  |  | 0.87 |
| DBD | 57 (60) | 39 (61) |  |
| DCD | 37 (40) | 25 (39) |  |
| ECD | 81 (87) | 53 (82) | 0.34 |
| CIT (h) | 14.92 ± 6.02 | 14.45 ± 4.33 | 0.59 |
| Induction IS |  |  | 0.21 |
| Thymoglobulin | 62 (66) | 48 (75) |  |
| Basiliximab | 32 (34) | 16 (25) |  |
| Maintenance IS |  |  | 0.62 |
| PDN + TAC + MMF | 38 (40) | 28 (44) |  |
| PDN + TAC + mTORi | 56 (60) | 36 (56) |  |

Data are expressed as mean ± SD, median [IQR] or n (%) unless otherwise indicated. KT, kidney transplantation; BMI, Body Mass Index; DM, Diabetes Mellitus; IHD, Ischemic Heart Disease; CVA, Cerebrovascular Accident; HLA, Human Leukocyte Antigen; DDKT, Deceased Donor Kidney Transplantation; DBD, Donor after Brain Death; DCD, Donor after Circulatory Death; ECD, Expanded Criteria Donor; CIT, Cold Ischemia Time; IS, immunosuppression; PDN, Prednisone; TAC, Tacrolimus; MMF, mycophenolate; mTORi, mTOR inhibitor.

Table S3 - Causes of recipient death in both age groups

|  | 60-65 years old  n (%) | ≥ 75 years old  n (%) | p |
| --- | --- | --- | --- |
| Infection | 4 (33) | 8 (73) | 0.14 |
| Cardiovascular disease | 3 (26) | 0 (0) |  |
| Neoplasia | 1 (8) | 0 (0) |  |
| Other causes | 0 (0) | 1 (9) |  |
| Unknown | 4 (33) | 2 (18) |  |
